# Family Physician’s and Primary Care Team’s Perspectives on Supporting Family Caregivers in Primary Care Networks

**DOI:** 10.3390/ijerph18063293

**Published:** 2021-03-23

**Authors:** Jasneet Parmar, Sharon Anderson, Marjan Abbasi, Saeed Ahmadinejad, Karenn Chan, Lesley Charles, Bonnie Dobbs, Amandeep Sheny Khera, Jennifer Stickney-Lee, Peter George J. Tian, Suvidha Jain

**Affiliations:** Department of Family Medicine, Faculty of Medicine and Dentistry University of Alberta, Edmonton, AB T6G 2T4, Canada; jasneet.parmar@albertahealthservices.ca (J.P.); mabbasi@ualberta.ca (M.A.); ahmadine@ualberta.ca (S.A.); kchan1@ualberta.ca (K.C.); lcharles@ualberta.ca (L.C.); bdobbs@ualberta.ca (B.D.); skhera@ualberta.ca (A.S.K.); Jennifer.Stickney-Lee@albertahealthservices.ca (J.S.-L.); peter.tian@ualberta.ca (P.G.J.T.); suvidha@ualberta.ca (S.J.)

**Keywords:** family physicians, primary care, family caregiver, carers, chronic disease, multidisciplinary team

## Abstract

Background. Research, practice, and policy have focused on educating family caregivers to sustain care but failed to equip healthcare providers to effectively support family caregivers. Family physicians are well-positioned to care for family caregivers. Methods. We adopted an interpretive description design to explore family physicians and primary care team members’ perceptions of their current and recommended practices for supporting family caregivers. We conducted focus groups with family physicians and their primary care team members. Results. Ten physicians and 42 team members participated. We identified three major themes. “Family physicians and primary care teams can be a valuable source of support for family caregivers” highlighted these primary care team members’ broad recognition of the need to support family caregiver’s health. “What stands in the way” spoke to the barriers in current practices that precluded supporting family caregivers. Primary care teams recommended, “A structured approach may be a way forward.” Conclusion. A plethora of research and policy documents recommend proactive, consistent support for family caregivers, yet comprehensive caregiver support policy remains elusive. The continuity of care makes primary care an ideal setting to support family caregivers. Now policy-makers must develop consistent protocols to assess, and care for family caregivers in primary care.

## 1. Introduction

Family caregivers are critical partners in chronic disease management. They provide 70 to 90% of the care to persons living with frailty, complex chronic conditions, and impairments in the community [1,2,3] and are considered “key partners in long-term care” [4,5]. We define family caregivers as any person who takes on a generally unpaid caring role and provides emotional, physical, or practical support in response to physical and/or mental illnesses, disabilities, or age-related needs. Family caregiving becomes more onerous as chronic illnesses, frailty, and impairments progress. In the last two decades, medical advances, increased longevity, shorter hospital stays, as well as the shift to community care have made family caregiving more complex and longer-lasting. [1,6] At the same time, demographic changes, family structure, and gendered role changes, and health and continuing care practice reforms have increased the demand for care while threatening the supply of family caregivers. [6,7,8] Despite this, family caregivers have not abandoned the caregiving role. However, they are dealing with heavier care workloads and longer care trajectories [1,3,9].

Caregiving takes a toll on family caregivers’ wellbeing [1,3,9]. Compared to non-caregivers, family caregivers have higher rates of depressive symptoms, anxiety, stress, and emotional difficulties than their non-caregiving counterparts [1,3,10,11]. Caregiver distress is rising. In 2016, over a third (33.3%) of family caregivers to homecare care recipients in Canada were stressed and distressed [7,12], rising from 15.6% in 2010 [13]. The rates of family caregiver distress are even higher since the COVID-19 pandemic began [14,15]. It is critical to note that it is not caregiving per se that is stressful, rather it is being overloaded with care work [1,16]. The family caregivers at the greatest risk of distress and premature mortality are those who live with the care-recipient, provide more than 20 h of care weekly, and care for a person with moderate to severe impairments (functional, cognitive), dementia, depression, and/or responsive behaviors [1,17,18].

Family physicians and their primary care teams are well-positioned to identify all family caregivers, assess their health, and refer them to supports they need throughout their particular care trajectory [19,20]. In fact, primary care is noted for health-promoting benefits of such continuity of care [21,22]. Globally, however, research, practice, and policy have focused on educating family caregivers to care but failed to equip the healthcare workforce to effectively support family caregivers [8,23,24]. Family caregivers are often marginalized by a healthcare system that focuses exclusively on patients and safeguarding their health, autonomy, and privacy [7,25,26,27,28]. Most healthcare providers do not assess family caregivers’ health, skills, or willingness to provide care, nor do they provide family caregivers with emotional support or assistance to effectively navigate health and community systems and access resources [7,8].

In response, governments have begun to enact health policies to support family caregivers. In the 2014 Care and Children and Families Acts, the United Kingdom mandated general practitioners to identify and assess caregiver’s needs [29,30]. The United States followed in January 2018 with the bipartisan Recognize, Assist, Include, Support, and Engage [RAISE] Act. RAISE directs the Department of Health and Human Services to develop and maintain a strategy for healthcare providers to recognize and support family caregivers [31].

As health providers and policy-makers move toward policies to support family caregivers, it is important to understand family physicians’ and primary care team members’ perceptions of their roles in supporting family caregivers. With this goal in mind, we studied perceptions of their current roles in supporting family caregivers, if they felt primary care was equipped to support family caregivers, what they might need to support family caregivers and their recommendations for supporting family caregivers going forward.

## 2. Materials and Methods

In this qualitative study, we utilized Thorne’s [32,33] qualitative interpretive description methodology to examine how individuals make sense of everyday practices. Interpretive description approaches begin with an investigation of what is known and not known empirically about healthcare practices with a relatively small purposive sample. Health professionals from three diverse primary care networks, an inner-city, urban, and suburban/rural, participated in the study. Primary Care Networks comprise groups of family physicians working with other health care professionals such as nurses, nurse practitioners, dietitians, pharmacists, social workers, and mental health professionals. Each primary care network puts together a team and develops services to meet the needs of their local community and the health issues within their practices.

### 2.1. Data Collection

After receiving ethics approval from the University of Alberta Health Ethics Research Board and informed written consent for the interviews and audio recordings from participants, we conducted four focus groups, one with each of three primary care teams and then one with family physicians. We reasoned that that separating physicians and teams might enable both to speak freely about everyday practices with family caregivers. Logistically, it was also easier to schedule the staff interviews at lunchtime and physician’s interview in the evening. The team focus groups were completed first. We utilized a semi-structured interview guide. A semi-structured interview guide enables the interviewer to ask follow-up questions about the participant’s responses, in particular, why, (e.g., why a practice was adopted or not adopted) or how (e.g., How did that work in your practice?) (See Table 1 Semi-structured interview questions).

Team focus groups ranged in length from 52 to 60 min and the physicians’ focus group was 90 min long. The focus groups were facilitated by an interviewer trained in qualitative interviewing [34]. The digitally recorded interviews were professionally transcribed, checked for accuracy, and cleaned to remove all identifying information. The cleaned transcripts were imported into NVivo for ease of data management.

### 2.2. Data Analysis

In accordance with Thorne’s [32,33] emphasis on careful analysis when using interpretive description methodology, we chose to use Braun and Clarke’s thematic data analysis methods [33,35]. Thematic analysis is a flexible qualitative method used to explore the different perspectives held by research participants, highlight the similarities and divergences in their viewpoints, and then generate thematic insights [33,35].We methodically followed Braun and Clarke’s [33,35] six stages of analysis (See Table 2, Stages of Thematic Analysis). A qualitative researcher (SA) and family physician resident (SJ), both members of the research team, independently read the transcripts to become familiar with the data and generate first impressions of meaning (Stage one). They made notes of impressions on the word transcripts. They discussed the initial impressions, then imported the data into NVivo. In stage two, two researchers (SA, SJ) worked separately to inductively generate initial open codes (*n* = 373). Then in stage three, two team members (SA, SJ) worked together to generate categories (*n* = 14). They identified patterns within the open codes and grouped codes with similar attributes and meanings. The principal investigator (JP) and two coders (SA, SJ) met to refine the categories into preliminary themes (Stage four) At this stage, discussing how the knowledge might apply in clinical practice and teamwork was useful to refining the themes (*n* = 3). SA and SJ reread the transcripts to verify and name the final themes (Stage five). The report was generated (Stage six) and discussed at a final team meeting. In this paper, we place the physicians’ and their primary care teams’ side by side in a table to illustrate the similarity in their responses. Direct quotes are used illustrate participants’ viewpoints.

## 3. Results

There were 52 participants, ten physicians, and 42 primary care team members from three primary care networks. The team focus groups were almost equally proportioned (*n* = 13, 14, 15) and participants were primarily registered or licensed practical nurses. Each team had one social worker in attendance and then a variety of other professionals because each primary care network employs staff to serve the specific needs of their population. The majority were female See Table 3: Participant demographics.

### 3.1. Themes

We identified three themes. Theme 1 “Family physicians and primary care teams can be a valuable source of support for family caregivers” acknowledges physicians and primary care teams’ broad recognition of the need to support family caregivers to sustain care and maintain health and wellbeing. Theme 2, “What stands in the way” Barriers to supporting family caregivers in primary care”, relates to the current barriers to supporting family in primary care practices. The patient-focused health system, problems identifying family caregivers, lack of a formal system for family caregiver assessments, and difficulties assisting caregivers to navigate resources prevented them from providing systematic support to family caregivers. Finally, Theme 3 “A structured approach may be a way forward: “proposals to support family caregivers, speaks to participants’ visions of how family caregivers might be supported in primary care. They proposed protocols for three tasks: identifying family caregivers; assessing family caregiver needs, and; assisting family caregivers to navigate resources that could be implemented in primary care. See Table 4: Quotes illustrating all themes.

#### 3.1.1. Theme 1: Family Physicians and Primary Care Teams Can Be a Valuable Source of Support for Family Caregivers

Family physicians and their primary care teams were very cognizant of the value of family caregivers’ roles in supporting their patients and were sympathetic to their stresses. There was broad agreement that the family physician and primary care team could be a source of ongoing support throughout care trajectories.

*Good for the patient too.* Family physicians and care teams noted that family caregivers were critical partners in supporting their patient’s wellbeing. Several times throughout the focus groups, physicians and the teams reiterated that if the caregivers were stressed and over-burdened with care work, the caregiver’s wellbeing would suffer and that jeopardized patient care. Physicians stressed that their knowledge of the patient and family caregiver was particularly useful in transitions from hospital to home or in situations where the patient’s care needs increased.

*Continuity.* Family physicians and the primary care teams positioned the continuity of care offered in primary care as the major benefit to family caregivers. They reported that they were identifying family caregivers, making time to ask what the family caregivers needed, and helping family caregivers to access resources. As the primary ongoing health system contact, even when family caregivers did not identify as a caregiver or refused their recommendations, they could continue to monitor the caregiver’s health and offer support in future appointments.

#### 3.1.2. Theme 2: “What Stands in the Way” Barriers to Supporting Family Caregivers in Primary Care

This theme focused on the barriers to supporting caregivers within these primary care networks. Family physicians and their primary care teams’ efforts were hampered by the patient-focused health system, caregivers not identifying as family caregivers, delayed identification of family caregivers in crisis, onerous navigation, and lack of transportation for family caregivers.

*Family caregivers are not patients.* The key problem was the patient-focused health system. While physicians and their teams could offer services to patients on-record in the primary care network, they were limited in, and were unsure of what they could provide to family caregivers who were not their patients. Physicians reported gingerly asking caregivers if they had a family doctor and being particularly challenged when family caregivers disclosed not having a family doctor. They recognized care from a family physician would be beneficial for family caregivers but in these busy practices, family physicians lacked space in their patient panel to accept the family caregiver as an additional patient. Physicians and teams reported that supporting family caregivers was time-consuming and there was little reimbursement for time-consuming conversations with non-patient family caregivers.

*Caregivers do not identify as family caregivers.* Unlike the patient who has a clear role identity in primary care, all participants noted identifying family caregivers was difficult. Participants observed that family caregivers who accompanied the patient to appointments viewed themselves as spouses, sons, or daughters, rather than as family caregivers. Large families in which there were multiple caregivers made it challenging for physicians and primary care teams to determine if there was a primary family caregiver and who that family member was. Participants were particularly challenged to identify caregivers when family members disagreed with one other.

Once family caregivers were identified, there was not an ideal spot to document the family caregiver(s) in the records. Physicians noted that a crisis, stress, or transition from hospital often precipitated identification and possibly an assessment of the primary family caregiver’s immediate needs to provide care, but not necessarily of the family caregiver’s own needs or willingness to care.

*Assessments were informal.* Physicians and primary care team members stated casual conversations with caregivers were the primary method by which they assessed caregiver needs. Stress or a crisis often triggered a deeper dive, and most often stressed family caregivers were referred to the in-house primary care network mental health team for assessments. One primary care team was using a screening tool for caregiver stress/ burden and the two others had assessment tools as part of pilot projects but noted no one tool had been transferred into routine practice.

*Onerous navigation.* Physicians and the primary care teams were frustrated by the complexity of navigation. They charged it was onerous for patients, caregivers, and for them to find person-centered supports and services. They recognized that family caregivers had diverse needs and required supports tailored to their current situation. Physicians said they did not have time to do a comprehensive needs assessment nor assist family caregivers to navigate to customized supports. They preferred to refer family caregivers to a trained team member who would link the caregiver to supports. All participants stressed navigation was challenging and time-consuming because often referrals were to services and organizations outside of the health systems. Within each of the three primary care networks, and then in individual practices within the networks, team members were assembling lists of community and health services.

Staff participants from all three teams referred to Google being their best search option. They noted that generally there were fewer services for family caregivers than for patients both in-house and in the community. Moreover, they reported that community services for caregivers often disappeared or services were reduced due to a lack of sustainable funding. It was particularly time-consuming to find appropriate resources for family caregivers with unique needs (e.g., family caregivers to young adults aging out of pediatric services, rare conditions, young-onset stroke/dementia).

Participants agreed that access to affordable, accessible transportation was a significant navigation barrier as well as to family caregiver’s use of the services. Older adult caregivers were reluctant to drive far from their homes and working caregivers found taking time off work to drive care-receivers to appointments was time-consuming. The suburban team noted that transportation was a greater barrier to their rural than urban family caregivers because rural caregivers were often reluctant to drive in busy traffic in unfamiliar urban areas. The longer distances with increased transportation costs and time were also greater barriers for rural caregivers.

#### 3.1.3. Theme 3: “A Structured Approach May Be a Way forward.” Proposals to Support Family Caregivers in Primary Care

“A structured approach as the way forward” focuses on how physicians and their primary care teams thought family caregivers should be supported in primary care. Physicians and primary care teams proposed a person-centered team approach that would begin with the physician or a team member identifying family caregivers followed by an assessment of their needs, then a “warm handoff” to appropriate health and community systems resources.

*Assessment and navigation.* The participants stressed that diversity within family caregivers, the care-receiver’s needs, care trajectories, and living situations necessitated a person-centered approach. They suggested that a family caregiver required personalized assessments of their stress, goals, and support needs, followed by navigation to supports matched to the family caregiver’s needs. They stressed that family caregivers focused on the patient’s needs, yet they also needed support to maintain their own health. Some physicians noted they currently referred family caregivers who were patients in the practice primarily to home care and the services within their primary care network (e.g., mental health team, group education such as stress reduction exercise, nutrition). They referred both patients and non-patient family caregivers to caregiver support or disease-specific not-for-profit organizations (Caregivers, Alzheimer’s, Parkinson’s, asthma).

Staff from one primary care team reported that in-house patient referrals to members of their geriatric or mental health teams triggered an assessment of the family caregiver’s burden and support needs followed by a person-centered care plan. Team members stressed that it was easier to refer to resources within their network and the health system. As well, within the health system, they received acknowledgment of the referral and often a follow-up report. Staff were uneasy about the lack of similar healthcare reporting standards in handoffs to community organizations.

*Navigation needs a warm handoff:* One physician proposed the development of a widely available searchable database mapped to caregiver’s support needs. Other physicians and the teams reported that several good lists were available, but many family caregivers and patients required a knowledgeable person to help them access supports. Physicians and their primary care teams endorsed a model in which navigators or home care case managers with knowledge of the family caregiver’s situation and needs, as well as knowledge of the health and community resources, could provide more person-centered supports. They also recommended linking the navigator or homecare case manager to the primary care network or even locating them within physicians’ offices to encourage teamwork.

*Spread and scale of best practices.* Participants acknowledged that they were developing best practices to support family caregivers within their networks and wanted that knowledge disseminated throughout primary care practices. All participants reported that their primary care network teams had participated in several research studies or pilot projects in which they had been introduced to tools to assess family caregivers’ stress or burden and protocols to support family caregivers. They observed that the way forward depended on ensuring primary care physicians are mandated and funded to care for family caregivers.

## 4. Discussion

We used interpretive description [32,33] to investigate family physicians and their primary care network team’s approaches to family caregivers. Interpretive description was designed to find out what is known and not known about healthcare practices with a purposive sample. This study follows a scoping review in which we found that collaborative primary care models were recommended as the most feasible model for supporting family caregivers yet there was little literature on family physicians’ perspectives on their roles in supporting family caregivers [36].

In this sample of three diverse primary care teams in one western Canadian province, there was evidence that physicians and their teams recognized that family caregivers need support throughout longer and very diverse care trajectories. They thought that primary care assessments earlier in the caregiving journey might alleviate later distress as care becomes more onerous. In the “structured approach may be a way forward” theme, participants recognized that a more systematic approach to family caregivers was preferable than the current ad hoc tactics. These family physicians and their primary care teams envisioned a strengthened role for primary care to support family caregivers within an integrated system of health and community supports. They portrayed their roles as identifying family caregivers, supporting their mental and physical health, and signposting them to appropriate services and supports. In fact, some teams were developing procedures within their primary care networks to support the family caregivers who were registered patients. These results reflect the increasing interest in healthcare providers’ role in supporting family caregivers [36,37,38].

The primary care physicians and team members participating in this research, like others worldwide, recognize that primary care can play a significant role in supporting family caregivers to provide the care their patients need as well as the help patients need to manage chronic conditions [30,38,39]. The comprehensiveness, continuity, and coordination available within primary care are particularly attractive in the management of care trajectories that can last for many years [38,40]. Indeed, primary care support results in better patient care, improved caregiver and provider communication, and validation of caregivers’ efforts [38,39]. However, caregivers have not been a priority in the patient-focused health system [41,42]. It was easier for these primary care teams to address the needs of patients who were family caregivers than non-patients who were accompanying the patient to an appointment,

Similar to our Canadian primary care participants, practitioners in the United States [US] [43] and health and social care providers, commissioners, and policy-makers in the United Kingdom [UK] [30] also thought that primary care was an ideal setting to identify family caregivers and begin the support process. As our Canadian primary healthcare providers reported, family caregiver assessments in the United States also tended to be informal [44]. Only 7.5% of practices and clinics had standardized caregiver assessment procedures. [44] Lack of time (81.1%) and inadequate reimbursement (39.6%) were the greatest barriers to caregiver assessments and supports. About a quarter wanted better navigation and referral options for family caregivers [44]. At present, no guidelines exist for caregiver assessments in primary care in the US [44] or Canada [40].

In this study, participants noted they are reimbursed for providing care to caregivers who are patients, but not for non-patients. Often interactions with family caregivers are time-consuming. General practitioners in the UK are mandated and reimbursed to identify family caregivers [45]. However, a 2015 study found UK family caregivers had a worse patient experience in primary care than non-caregivers [45]. The authors asserted that improving the primary care experience should be a strategic priority for clinicians and policy-makers but did not provide specific recommendations. Carduff and colleagues [46] suggested there is still ambiguity about the role of general practitioners and primary care teams in identifying and supporting carers. The UK primary care system remains patient-focused [30,46]. UK General practitioners are concerned about the lack of time to assess family caregivers’ needs and then being overwhelmed by the demand for caregiver servicers that may not be available [30,46]. Peters and colleagues [30] suggest that primary care’s ambivalence to identify family caregivers might be addressed by navigation and access to needed supports such as resources for patient care and respite for the caregiver.

The difficulty navigating disconnected health and community systems was a subtheme running through all three of our themes. Physicians and primary care teams recognized that family caregivers need assistance to access supports tailored to their situation and they had difficulty finding tailored resources. Taylor and Quesnel-Vallée [47] report that family caregivers in Canada and the United States now spend 15 to 50% of their time trying to find and access the services they need to sustain their caregiving and to maintain their own wellbeing.

One primary care team had developed a geriatric team approach in which nurses managed assessments and navigation, however, participants noted that all family caregivers would benefit from a systematic approach in primary care. They suggested linking a navigator or home care case manager to the primary care network to facilitate navigation. The UK is moving to a social prescribing model [29], in which a navigator or link worker receives the physician’s referral and works collaboratively with family caregivers to connect them to health and community services. It is not clear that tackling navigation will overcome the obstacles of the patient-focused system and the structural barriers such as insufficient time and reimbursement which are identified by primary care professionals as larger barriers to initiating consistent care for family caregivers [28,30,36,37,42].

Despite a plethora of research studies [48,49,50] and reports [51,52,53] recommending proactive, consistent support for family caregivers, comprehensive caregiver support policy remains elusive. The primary care professionals participating in our research acknowledged just how difficult it is for them to support family caregivers. New models such as Doctors of BC policy paper *“Circle of Care: Supporting Family Caregivers in BC”* [40] and the Manitoba Caregiver Recognition Act, [54] lay the groundwork for family caregiver assessment and support. Now policies, practice guidelines, reimbursement models, and educational curricula are needed to translate research and recommendations into practice [36]. Specifically, other studies have also recommended that ethics guidance [55,56] and billing codes are needed for work with family caregivers in primary care [42,44,46].

### Limitations

This study included only three primary care teams and their views are not necessarily the views of all primary care teams. The aim of the interpretive description study was to explore what teams were doing and how primary care might be part of the solution to support caregivers consistently. The health professionals participating in the group interviews were very aware of family caregivers’ care work and the impact of caregiving on their health. They could be outliers or trailblazer advocates for ensuring family caregivers are supported in primary care. However, recent research [30,38,43] concurs with our pragmatic sample that primary care professionals are supportive of a systematic approach to family caregivers in primary care.

## 5. Conclusions

The results show that family physicians and their primary care teams thought that primary care could support family caregivers consistently. They proposed roles in identifying family caregivers, assessing their needs, and signposting them to supports. However, it is not enough to recognize that primary care can be part of the solution to enable family caregivers to sustain care and maintain their wellbeing. The family caregiver role must be recognized in primary care mandates and reimbursement policies. Policy-makers must work with family physicians to develop consistent protocols to assess, engage, and support family caregivers.

## Figures and Tables

**Table 1:** Focus Group Semi-Structured Interview Guide.

Family Physicians and Primary Care Teams
How much do you interact with family caregivers in your practice? How do you see your role with family caregivers? Do you feel equipped to understand and care for the needs of family caregivers? How do you think you can support the needs of family caregivers here in primary care? Are there any barriers to supporting family caregivers in primary care? Do you think there is anything you can do professionally to better support family caregivers here in primary care?

**Table 2:** Stages of Thematic Analysis.

Braun and Clarke’s Six Phase Approach to Thematic Analysis
Stage 1: Become familiar with the data by listening to the recordings and reading through the transcripts Stage 2: Generate initial codes through an open coding process Stage 3: Search for themes from the open code to generate categories Stage 4 Review categories to propose preliminary themes Stage 5: Define and name final themes by comparing preliminary themes against raw data Stage 6: Generating a report

**Table 3:** Participant Demographics.

		Physicians	Primary Care Teams
Participants		10	42
Gender	Female	8	39
	Male	2	3
Age	Under 25	0	1
	25–34	3	13
	35–44	1	11
	45–54	4	15
	55–64	2	1
	Over 65	0	1
Professions	Family Physician	10	0
	Registered Nurse		23
	Psychiatric Nurse		1
	Licensed Practical Nurse		8
	Social Worker		3
	Respiratory Therapist		1
	Psychologist		1
	Exercise Physiologist		1
	Community Coordinator		2
	Dietician		2

**Table 4:** Direct Quotes Illustrating Themes.

Physicians	Primary Care Teams
**Theme 1: Family physicians and primary care teams can be a valuable source of support for family caregivers**	
Good for the patient too	
First, just by acknowledging they have a hard role, like, “Hey you’re here again with your mom or whoever.” And I often ask them, “Do you have a family doctor of your own?” If the family caregiver is distressed, then that affects the patient [Doctor 8]	I think it is really important for us to identify family caregivers and especially burnout, because that affects the patient’s health as well. [PCT#2]
Primary Care continuity fosters relationship and trust	
The therapeutic alliance. Even though that might not be with the caregivers, but it does foster confidence and a better relationship, over-time right? [Doctor 10] Family physicians and primary care teams could be a valuable source of support for all family caregivers. I think we are getting better at doing that. [Doctor 5]	Because the patient usually comes back to our clinic, we do see them a lot, so you develop that trust with them. It happens over-time. Like it doesn’t in an initial on the first visit. Sometimes it’s not identifiable or it takes time. [PCT#2]
**Theme 2: “What stands in the way” Barriers to Supporting Family Caregivers in Primary Care**	
Patient-focused health system	
The ones that I know are in a more demanding position, I’ll ask them to always book an appointment for themselves around that same time, or at least every couple months, I do that, just so we can touch base on how they’re doing or follow-up on some of their health concerns that often get pushed aside. So, it only really works if they’re your own patient, but some of them I’ve gotten into a habit of doing that every three months. We’ll just have them both together, and then that way you have a bit more time and you can kind of shuffle the time to the one or the other that needs it as well. [Physician 3]	Really, we are there to support the patient. We support the caregiver indirectly unless they are our patients and then we can support them directly. So yes, we refer them within the network, and we have lots of services within the network and we also refer them to Caregivers Alberta or the Alzheimer’s Society. [PCT#1] It’s a lot easier if you’re working with the caregiver and they are your patient and that the problems that they’re presenting or the thing they’re asking for help with is their own caregiver burden and their own stress. We have social workers, nurses, and a mental health team you can talk to. [PCT#3]
Patient-focused health system-- Ethically challenged when family caregivers don’t have family physicians	
People don’t have a family doctor, and they’re busy, and they’re not taking care of themselves by seeing their family doctor if they have one. But if they don’t have one then it becomes really difficult because if you know this person needs to see a physician, then we’re thinking like, “Oh my God, do I try to add him onto my panel as well?” knowing that it will help your patient be taken better care of if the health needs of the caregivers, if they’ve got their doctor. The logistics of primary care, and well, healthcare and community systems stand in the way of effective support for family caregivers [Physician 5]	It is really hard when they aren’t patients, and they don’t seem to have a family doctor. We can tell them about the Alzheimer’s Society or Caregivers Alberta, but really, they need that regular care. [PCT#2] You see them talking to other caregivers in the waiting room, but it is not really our role to ask them if they need help or to refer them to services. They are not our patients. [PCT#3}
Family caregivers don’t identify	
Sometimes, to be honest with you, the primary caregiver hasn’t self-identified as the caregiver. [Physician 7] Yeah, I think part of the trouble is that when there’s large families, sometimes you communicate to one, but there’s a lot of families where these two siblings don’t talk, or this person was somewhere else. And sometimes we end up communicating the plan to two or three people in one day, even. [Physician 3]	Like I’m not a caregiver, I’m just helping or whatever. Just doing my job but caring 24/7 doing all of the care and the housework, mowing the lawn, shoveling the oh my God. But no, I am not a caregiver. [PCT#1] I feel like there’s parts that caregivers don’t fully acknowledge maybe the load that they’re actually carrying. I also feel it needs to be normalized for people to feel comfortable to acknowledge that because you see them come in with the patient and you know that they’re probably doing 85% of the things that their spouse or whoever needs, but they just do it. [PCT#2]
But there’s no ideal system for recording the caregiver in the patient chart or to link them. Perhaps NetCare or a substitute of it would let you identify patients instead of as patients but as family units. And they can link each other’s identifications. [Physician 4]	On NetCare, often you can look up next of kin for urgency visits [Speaker 5]. Find out who’s on their list for their contact [Speaker 4]. Sometimes I just pop-up notes to look for the person that looks after the person or we could discuss it with the person. I think we are working on some other ways of identifying, but there are not any in place yet. [Speaker 5] PCT#1,
Crisis triggers assessment	
Especially in a primary care setting, because a crisis may not have yet happened, they may not have identified or been assessed. In a hospital, you’re like, “Okay, who’s onboard? You’re designated, you’re the primary caregiver.” But when you’re in a primary care environment where you’re literally coming from your home, your street environment back into the clinic, you may not see yourself as that caregiver. So I think it is having that conversation. [Physician 7]	Or they verbally tell you, “I’m very stressed. I can’t do this anymore. I don’t know what to do.” Those are kind of the things that I look for. [PCT#2] I know our mental health team can get called in. Sometimes they’re not referred because they haven’t been identified and then we see them and we realize that they’re basically burnt out from stress. [PCT#3]
Assessments were Informal	
If they’re my patient I think I would assess them, mostly for the purpose of knowing how their wellbeing is, as you would if you saw any of your patients. But if they weren’t your patient I think my goal as far as saying, “How are you doing?” And maybe they elaborate very much on it and it won’t even be anything medical. Especially if they’re not even affiliated with your clinic, you may have to review directly up for further assessment. [Physician 3] There is not a very structured way that we support our family caregivers. I can think of a lot of little piecemeal examples, but it doesn’t have any kind of formalized structure really. [Physician 10]	I think when she says she does it routinely, so we have different disciplines and they’re each working within different programs, and so our senior’s community hub. Two nurses down there are part of that program, so that will be part of their regular assessment. [PCT#1] There’s no specific screening tool, but once we’ve identified that there are stressors in certain areas, then we can break it down and help them develop some goals to spend more time with self-care and identify with that, as a priority would look like. But no specific checkbox screen. But, I think a good Spidey sense, also curiosity, so knowing when to dig deeper. The Spidey sense leads you to ask more questions than just leave. [PCT#3]
Onerous Navigation.	
Yeah, everybody is keeping these repositories of resources by themselves, some in your head, some are on bulletin boards, and some are in your drawer. [Doctor 5] I think it puts us in a bit of an awkward position because we’re trying to encourage them to go-to these places, but that’s a huge piece missing in that puzzle that they can’t get there because they don’t drive that far or the service was there and can’t continue because of funding cuts. [Physician 10]	Speaker 5: Well, my experience with 211, using them for various other things, is that the people who are answering the phone don’t know enough about the programs to actually do that navigation. They’re working off a screen. But it isn’t really an experienced person who really knows those programs. So sometimes it’s just a waste of time. I could Google it faster probably. [PCT#1] I have to say it’s extremely time-consuming to make sure that what we have on there is current, because I’ll just give you an example, we partnered with Eldercare for a while last year and they were hosting character support groups here on site and then they lost their funding. [PCT#3]
Transportation	
Well, many health outcomes are driven by community services like housing, income, or transportation. So transportation is a problem and these organizations like Caregivers Alberta or Alzheimer’s Society are very caregiver focused, and they actually can walk the caregivers through getting benefits, getting transportation, getting applications made for income supplements, and stuff like that. [Physician 8]	No, transportation’s a huge problem. It ties into finances a lot of the times but it also ties into the idea of respite for the caregiver. If an individual who is caregiving is also the main source of rides and transportation, and their time requirement has gone up, but it also decreases the independence of the carer if they can’t access transportation. [PCT#2]
**Theme 3 “A structured approach may be a way forward.” Proposals to support family caregivers in primary Care**	
Assessment and Navigation	
When you have a book with a resource then it almost again takes the conversation away. Because what you need is a better understanding. I think what we need is recognizing that we can have these conversations. And then support to find the content from the conversation. What are some of the things that need to be designed there? Maybe that’s a go-to person who’s helping you with that, or it is some kind of smart pathway in or something. I agree too that I don’t have the time, just by nature of the way we practice. The doctor is not necessarily the person to be eliciting all that, just to start the conversation. [Physician 7]	Speaker 4: The hand holding, the warm handoff, and then someone who gets back to us and lets us know where they are at… that’s missing. Often, we don’t hear from the caregiver and you don’t hear from the place you sent them. [PCN1] If they’re interested in additional stuff or learning about the respite options or the programming that comes with respite or caring at the location, that connection kind of gets made in a bit more of a warm transfer rather than just saying, “Here’s the number,” because we all know not everyone is going to be at that stage to respond to that information or act on it in the moment. [PCT#2]
Navigation Needs a Warm Handoff	
I think what would be good is if you had some kind of program where you could match a patient or caregiver who’s at risk to have a comprehensive assessment, which would include an occupational therapist who assesses their home care needs. And then if they have any, they will give the home care number. Would they benefit from Meals on Wheels? Do they need any equipment? If so, here’s the number on how to get it. Physio, transition coordinator would be involved. If everybody could be under one roof in the PCN, and that could be one visit even, potentially. And then you match the resources to what is needed, rather than having this five-page booklet of, “Here’s what’s out there, good luck.” [Physician 3]	Speaker 6: We need to strengthen the relationship between home care case managers and Primary Care because they often see that the work they’re doing with the family in their home as very separate, and they don’t need the Primary Care physicians involved in the care of this patient. Speaker 4: Even before you get the home care team, it’s just that essential intakes to create that barrier to care. There needs to be a warm handoff, where they’re accepting of our referrals. They need to work collaboratively with us not at arm’s length. [PCT#3]
Spread and scale of best practices	
The same thing with down at our Senior’s Team. Our whole idea was how can we elevate our PCN and mobilize team members to help provide us the support for vulnerable seniors who have caregivers? Identify a timeframe that caregivers emerge and see themselves as caregivers. Even close to seeing themselves as caregivers. And so yes, we trained the team and the team’s fantastic. Their knowledge has gone up, their awareness of things has gone up, but we’re still running into that same problem of disseminating that information. Now you have this elite team that functions well, but there’s still the rest of the 35 family practices with seven, eight doctors within our practices, still a ways from that. So how do you continue to share that, so they have ongoing learnings. And so, what we’re thinking our next step now is let’s go-to the source. The content holders are the caregivers and the patients. [Doctor 6]	We have done so many pilot projects, and they are helpful for caregivers and patients. They just don’t stick, because they are research or pilot projects. [PCT#3] A lot of the supports that they need are not given in healthcare and we know should they be. Healthcare can’t do everything but we can be really well informed about what is available and how do they go about accessing it. It is just not systematic. [PCT #2]

## Data Availability

Data available from authors.
